# Supplementary material for: Effects of sleep quality on non-alcoholic fatty liver disease: a cross-sectional survey
Source: BMJ Open. 2020 Oct 29;10(10):e039947. doi: 10.1136/bmjopen-2020-039947 (PMC7597499; doi:10.1136/bmjopen-2020-039947)
Supplement: Author's manuscript [file bmjopen-2020-039947.draft_revisions.pdf]

BMJ Open is committed to open peer review. As part of this commitment we make the peer review history of every article we publish publicly available.

When an article is published we post the peer reviewers' comments and the authors' responses online. We also post the versions of the paper that were used during peer review. These are the versions that the peer review comments apply to.

The versions of the paper that follow are the versions that were submitted during the peer review process. They are not the versions of record or the final published versions. They should not be cited or distributed as the published version of this manuscript.

BMJ Open is an open access journal and the full, final, typeset and author-corrected version of record of the manuscript is available on our site with no access controls, subscription charges or pay-per-view fees (http://bmjopen.bmj.com).

If you have any questions on BMJ Open's open peer review process please email info.bmjopen@bmj.com

BMJ Open

Influence of sleep quality on non-alcoholic fatty liver disease : a cross-sectional survey

Journal:	*BMJ Open*
Manuscript ID	bmjopen-2020-039947
Article Type:	Original research
Date Submitted by the Author:	30-Apr-2020
Complete List of Authors:	Takahashi, Atsushi; Fukushima Medical University School of Medicine, Gastroenterology<br>Anzai, Yukio; Watari Hospital, Gastroenterology<br>Kuroda, Masahito ; Fukushima Red Cross Hospital, Gastroenterology<br>Kokubun, Masae ; Jusendo Hospital, total medical checkup<br>Kondo, Yuichiro ; Fujita General Hospital, Gastroenterology<br>Ogata, Takashi ; Masu Memorial Hospital, Gastroenterology<br>Fujita , Masashi; Fukushima Medical University School of Medicine, Gastroenterology<br>Hayashi, Manabu ; Fukushima Medical University School of Medicine, Gastroenterology<br>Imaizumi, Hiromichi ; Fukushima Medical University School of Medicine, Gastroenterology<br>Abe, Kazumichi ; Fukushima Medical University School of Medicine, Gastroenterology<br>Tanji, Nobuo; Watari Hospital, Gastroenterology<br>Ohira, Hiromasa; Fukushima Medical University School of Medicine, Gastroenterology
Keywords:	Gastroenterology < INTERNAL MEDICINE, Hepatobiliary disease < GASTROENTEROLOGY, SLEEP MEDICINE

SCHOLARONE™
Manuscripts

I, the Submitting Author has the right to grant and does grant on behalf of all authors of the Work (as defined in the below author licence), an exclusive licence and/or a non-exclusive licence for contributions from authors who are: i) UK Crown employees; ii) where BMJ has agreed a CC-BY licence shall apply, and/or iii) in accordance with the terms applicable for US Federal Government officers or employees acting as part of their official duties; on a worldwide, perpetual, irrevocable, royalty-free basis to BMJ Publishing Group Ltd ("BMJ") its licensees and where the relevant Journal is co-owned by BMJ to the co-owners of the Journal, to publish the Work in this journal and any other BMJ products and to exploit all rights, as set out in our licence.

The Submitting Author accepts and understands that any supply made under these terms is made by BMJ to the Submitting Author unless you are acting as an employee on behalf of your employer or a postgraduate student of an affiliated institution which is paying any applicable article publishing charge ("APC") for Open Access articles. Where the Submitting Author wishes to make the Work available on an Open Access basis (and intends to pay the relevant APC), the terms of reuse of such Open Access shall be governed by a Creative Commons licence – details of these licences and which Creative Commons licence will apply to this Work are set out in our licence referred to above.

Other than as permitted in any relevant BMJ Author's Self Archiving Policies, I confirm this Work has not been accepted for publication elsewhere, is not being considered for publication elsewhere and does not duplicate material already published. I confirm all authors consent to publication of this Work and authorise the granting of this licence.

For peer review only

Influence of sleep quality on non-alcoholic fatty liver disease : a cross-sectional survey

Atsushi Takahashi[1], Yukio Anzai[2], Masahito Kuroda[3], Masae Kokubun[4], Yuichiro Kondo[5], Takashi Ogata[6], Fujita Masashi[1], Manabu Hayashi[1], Hiromichi Imaizumi[1], Kazumichi Abe[1], Nobuo Tanji[2], Hiromasa Ohira[1].

[1]Departments of Gastroenterology, Fukushima Medical University School of Medicine, Fukushima, Japan.[2]Department of Gastroenterology, Watari Hospital, Fukushima, Japan, [3]Department of Gastroenterology, Fukushima Red Cross Hospital, Fukushima, Japan, [4]Institution for total medical checkup, Jusendo Clinic, Koriyama, Japan, [5]Department of Gastroenterology, Fujita General Hospital, Date-gun, Japan, [6]Department of Gastroenterology, Masu Memorial Hospital, Nihonmatsu, Japan,

Correspondence to: Atsushi Takahashi, M.D.

Department of Gastroenterology, Fukushima Medical University, 1 Hikarigaoka, Fukushima 960-1295, Japan

E-mail: junior@fmu.ac.jp

Telephone: +81-24-547-1202; Fax: +81-24-547-2005

For peer review only

For peer review only - http://bmjopen.bmj.com/site/about/guidelines.xhtml

Word count: 2376

Abstract

Background The effects of sleep quality on the risk for developing non-alcoholic fatty liver disease (NAFLD) remain uncertain. Our purpose was to elucidate the association between sleep quality and NAFLD.

Methods We analyzed 4,828 participants who underwent health check-ups at 4 hospitals. Sleep quality was evaluated by the Pittsburgh Sleep Quality Index (PSQI), which comprised 7 elements that were scored from 0 to 3. We compared the global PSQI score and the score for each element between NAFLD and non-NAFLD separately by sex. Logistic regression analysis was performed to determine the association between NAFLD and each PSQI score.

Results In both men and women, the mean of the PSQI score for sleep medication use was significantly higher in those with non-NAFLD than in those with NAFLD. With regard to sleep medication use in men, the odds ratio (OR) (95% confidence interval (CI)) for NAFLD was lower with a score of 3 (OR0.60, 95% CI 0.38–0.95) than with a score of 0. The odds ratio for NAFLD based on daytime dysfunction was higher with a score of 3 than with a score of 0 in both men (OR 2.82, 95% CI 1.39–5.75) and women (OR 2. 08, 95% CI 1.10–3.92).

For peer review only - http://bmjopen.bmj.com/site/about/guidelines.xhtml

Conclusion Sleep medication use and daytime dysfunction were associated with NAFLD.

Key words: sleep quality, non-alcoholic fatty liver disease, sleep medication, daytime dysfunction

For peer review only

Strengths and limitations of this study

- The strength of this study lie in the sample size from multiple centers.
- This is the first study to use all 7 elements scores of the Pittsburgh Sleep Quality Index in the assessment of association between sleep quality and non-alcoholic fatty liver disease.
- The present study based on a self-reported questionnaire (Pittsburgh Sleep Quality Index), therefore, the associations between sleep quality and NAFLD need to be confirmed by objective methods.

Introduction

Non-alcoholic fatty liver disease (NAFLD) is the most common chronic liver disease, probably because of the widespread prevalence of unhealthy lifestyle habits, such as excess nutrition or less physical activities [1]. In patients with NAFLD, liver fibrosis has

For peer review only - http://bmjopen.bmj.com/site/about/guidelines.xhtml

been associated with the liver-related outcomes and mortality [2]. Lifestyle interventions, such as diet modification and exercise to achieve weight loss, are fundamental in the treatment of patients with NAFLD; in fact, weight loss of more than 7% to 10% has been reported to improve liver fibrosis in NAFLD patients [3-5]. Recently, the association of sleep disorder with NAFLD has attracted attention. Obstructive sleep apnea (OSA) and short sleep duration have been known to be associated with NAFLD [6-8], but only few have reported on the association between sleep quality and NAFLD [8-10].

The Pittsburgh Sleep Quality Index (PSQI) is an established and widely used method for sleep quality evaluation; it comprises 7 elements, including subjective sleep quality, sleep latency, sleep duration, habitual sleep efficiency, sleep disturbance, sleep medication use, and daytime dysfunction [11]. In previous reports on the sleep quality of patients with NAFLD, only the results of the global PSQI score or some components of the PSQI were assessed [8-10]. Therefore, the associations of all components of the PSQI with NAFLD are poorly understood. In the present study, we aimed to clarify the detailed association between sleep quality and NAFLD by analyzing all elements of the PSQI separately by sex and by adjusting for confounding factors.

Methods

For peer review only

For peer review only - http://bmjopen.bmj.com/site/about/guidelines.xhtml

Subjects

The subjects for this study comprised 6,138 Japanese men and women who underwent health check-ups from May 2013 to March 2014 at the Watari Hospital Health Center; Jusendo Hospital; Fujita General Hospital; Fukushima Red Cross Hospital; and Masu Memorial Hospital in Fukushima ken, Japan. We excluded 423 subjects who had insufficient data and 887 subjects who had chronic liver disease secondary to other causes (i.e., hepatitis C antibody-positive, hepatitis B surface antigen-positive, and alcoholic consumption >30 g/day for men and >20 g/day for women). NAFLD was defined as fatty liver detected by ultrasound in the absence of the above-mentioned causes of chronic liver disease. Accordingly, 4,828 subjects (1,864 men and 2,964 women) were eligible for this study. The study protocol was approved by the ethics committee of Fukushima Medical University School of Medicine (#1636). All patients provided written informed consent prior to study participation, and this study was conducted in conformity with the ethical guidelines of the Declaration of Helsinki.

Measurements

Before the examination, the subjects were asked to complete a questionnaire regarding their ordinary sleep using the PSQI scale and lifestyle factors, including smoking habits,

For peer review only - http://bmjopen.bmj.com/site/about/guidelines.xhtml

drinking habits, and physical activity. The PSQI scale comprised 19 self-administered questions that generated 7 elements, including subjective sleep quality, sleep latency, sleep duration, habitual sleep efficiency, sleep disturbance, sleep medication use, and daytime dysfunction. The score for each element ranged from 0 to 3, as follows: 1) subjective sleep quality (0 = very good, 1 = fairly good, 2 = fairly bad, and 3 = very bad; 2) sleep latency, which was defined as the required time in minutes to fall asleep each night and the frequency of falling asleep within 30 minutes; 3) sleep duration (0 for >7 hours, 1 for 6-≤7 hours, 2 for 5-<6 hours, and 3 for <5 hours); 4) habitual sleep efficiency, which was defined as the proportion of hours slept to hours spent in bed (0 for ≥85%, 1 for 75% to 84%, 2 for 65% to 74%, and 3 for <65%); 5) frequency of sleep disturbance, which was defined as the having trouble sleeping because of some reasons; 6) sleep medication use (0 for no use during the past month, 1 for less than once a week, 2 for once or twice a week, and 3 for three or more times a week; and 7) frequency of daytime dysfunction, which was defined as having trouble staying awake while driving, eating meals, or engaging in social activity and having trouble in sustaining enough enthusiasm to get things done. The cumulative score of these elements was defined as the global score, which ranged from 0 to 21. Poor sleepers were identified based on a global score of >5.

Physical activity was defined as walking or an equivalent amount of physical

For peer review only - http://bmjopen.bmj.com/site/about/guidelines.xhtml

activity for more than 1 hour a day. The subjects were examined after an overnight fast. Ultrasound was used to evaluate hepatic steatosis. An ultrasound diagnosis of fatty liver was defined as a bright liver; increased liver echogenicity, compared with that in the kidneys; vascular blurring; and deep attenuation of the liver.

Statistical analysis

All statistical analyses were conducted separately by sex, based on the fact that both sleep status and NAFLD have sex differences. The two groups (men and women) were compared using the Mann–Whitney U-test and the χ^2 test for categorical variables. For continuous variables, analysis of covariance was performed to determine any significant differences between the non-NAFLD and NAFLD groups, after adjustments for age, smoking habits, and physical exercise. After adjusting for the possible confounding factors, such as age, smoking habits, and physical activity, logistic regression analysis was conducted to assess the relationship of sleep disorder, based on the global PSQI score and the other 7 elements, with NAFLD. The values for odds ratio (OR) with 95% confidence interval (CI) were calculated from the logistic regression analysis. Statistical analyses were performed using SPSS 25.0 for Windows (SPSS, Inc., Chicago, IL, USA). A p value of <0.05 was considered significant.

For peer review only

For peer review only - http://bmjopen.bmj.com/site/about/guidelines.xhtml

Patients and Public involvement

Patients and public were not included in the present study. Patient consent is not required.

Results

Characteristics of the participants (Table 1)

The mean age was significantly higher in women than in men (55.5 years vs. 56.6 years; *p* = 0.002). Compared with men, women had significantly lower body mass index and sleep duration; less proportion of subjects with smoking history and NAFLD; higher level of physical activity; and significantly higher global PSQI score (*p* <0.001 for all).

Table 1 Characteristics of participants

	Men (n=1864)	Women (n=2964)	*p* value
Age	55.5 ± 12.7	56.6 ± 12.4	0.002
Body mass index kg/m^2	23.4 ±3.3	22.9 ± 3.5	< 0.001
Sleep duration min/day	388 ± 63	380 ± 59	< 0.001
Physical activity	41.5% (774)	47.1% (1395)	< 0.001
Current smoker	25.1% (467)	5.6% (165)	< 0.001
NAFLD	47.0% (876)	37.1% (1099)	< 0.001
ALT (U/L)	24.5 ± 15.5	20.7 ± 15.2	< 0.001
Global PSQI score	4.9 ± 2.6	5.2 ± 2.6	< 0.001

NAFLD; non-alcoholic fatty liver disease, *ALT*; alanine aminotransferase, *PSQI;* Pittsburgh sleep quality index

Comparison between non-NAFLD and NAFLD (Table 2)

For peer review only - http://bmjopen.bmj.com/site/about/guidelines.xhtml

The mean score for sleep medication use was significantly higher in those without NAFLD than in those with NAFLD in both men (0.21 ± 0.74 vs. 0.13 ± 0.59; $p = 0.044$) and women (0.28 ± 0.82 vs. 0.20 ± 0.70; $p = 0.030$). In men, the mean scores for sleep latency (0.58 ± 0.74 vs. 0.63 ± 0.78; $p = 0.066$) and daytime dysfunction (0.62 ± 0.72 vs. 0.71 ± 0.80; $p = 0.074$) tended to be lower in those without NAFLD than in those with NAFLD.

Table 2 Comparison of each PSQI score between the groups with and without NAFLD

	With NAFLD	Without NAFLD	*p* value
Men			
Global PSQI score	5.02 ± 2.65	4.85 ± 2.47	0.216
Subjective sleep quality	1.14 ± 0.61	1.07 ± 0.59	0.077
Sleep latency	0.63 ± 0.78	0.58 ± 0.74	0.066
Sleep duration	1.31 ± 0.81	1.27 ± 0.82	0.863
Habitual sleep efficiency	0.40 ± 0.66	0.40 ± 0.65	0.808
Sleep disturbance	0.70 ± 0.51	0.69 ± 0.50	0.414
Use of sleep medication	0.13 ± 0.59	0.21 ± 0.74	0.044
Daytime dysfunction	0.71 ± 0.80	0.62 ± 0.72	0.074
Women			
Global PSQI score	5.19 ± 2.59	5.29 ± 2.64	0.409
Subjective sleep quality	1.11 ± 0.60	1.11 ± 0.59	0.935
Sleep latency	0.72 ± 0.85	0.76 ± 0.84	0.443
Sleep duration	1.40 ± 0.77	1.41 ± 0.78	0.314
Habitual sleep efficiency	0.40 ± 0.63	0.40 ± 0.70	0.672
Sleep disturbance	0.69 ± 0.51	0.70 ± 0.50	0.947
Use of sleep medication	0.20 ± 0.70	0.28 ± 0.82	0.030
Daytime dysfunction	0.66 ± 0.73	0.63 ± 0.69	0.390

PSQI; Pittsburgh sleep quality index

Association between sleep disorder and NAFLD (Tables 3 and 4)

For peer review only - http://bmjopen.bmj.com/site/about/guidelines.xhtml

In men, the odds ratio for NAFLD was lower with a score of 3 for sleep medication use (OR0.60, 95%CI 0.38-0.95, p=0.030) than with a score of 0 (no sleep medication use); women had a similar tendency for the association between the score for sleep medication use and the odds ratio for NAFLD (OR0.75, 95%CI 0.54-1.04, p=0.084). The odds ratio for NAFLD was higher with a daytime dysfunction score of 3 than a score of 0 in both men (OR2.82, 95%CI 1.39-5.75, p=0.004) and women (OR2.08, 95%CI 1.10-3.92, p=0.024). In women, the odds ratio for NAFLD was lower with a score of 1 for sleep latency (OR0.82, 95%CI 0.69-0.97, p=0.022) than with a score of 0. On the other hand, the odds ratio for NAFLD was higher with a score of 1 for habitual sleep efficiency (OR1.29, 95%CI 1.29, p=0.004) than with a score of 0 in women. Our results showed no associations of NAFLD with the global PSQI score, subjective sleep quality score, sleep duration score, and sleep disturbance score.

For peer review only - http://bmjopen.bmj.com/site/about/guidelines.xhtml

Table3 Odds ratio of NAFLD by PSQI and its components in men (n=1864)

	Score	Number	Odds ratio	95% CI	*p* value
Global PSQI score	≤ 5	1202	1.00	reference	
	≥ 6	662	1.08	0.89-1.30	0.458
Subjective sleep quality	0	223	1.00	reference	
	1	1252	1.10	0.83-1.47	0.506
	2	363	1.24	0.89-1.75	0.207
	3	26	2.02	0.87-4.67	0.101
Sleep latency	0	1011	1.00	reference	
	1	624	1.05	0.86-1.29	0.622
	2	186	1.37	1.00-1.88	0.050
	3	43	1.31	0.70-2.43	0.398
Sleep duration	0	368	1.00	reference	
	1	656	0.99	0.76-1.28	0.923
	2	778	1.01	0.78-1.31	0.937
	3	62	1.04	0.60-1.80	0.894
Habitual sleep efficiency	0	1262	1.00	reference	
	1	500	1.00	0.81-1.23	0.991
	2	64	0.94	0.57-1.57	0.820
	3	38	1.23	0.64-2.37	0.534
Sleep disturbance	0	607	1.00	reference	
	1	1218	1.07	0.88-1.30	0.499
	2	38	1.13	0.58-2.19	0.726
	3	1	-	-	-
Use of sleep medication	0	1730	1.00	reference	
	1	26	0.97	0.44-2.12	0.939
	2	19	1.06	0.43-2.65	0.894
	3	89	0.60	0.38-0.95	0.030
Daytime dysfunction	0	912	1.00	reference	
	1	702	1.00	0.82-1.22	0.992
	2	210	1.11	0.82-1.50	0.520
	3	40	2.82	1.39-5.75	0.004

For peer review only - http://bmjopen.bmj.com/site/about/guidelines.xhtml

PSQI; Pittsburgh sleep quality index

Table4 Odds ratio of NAFLD by PSQI and its components in women (n=2964)

	Score	Number	Odds ratio	95% CI	*p* value
Global PSQI score	≤ 5	1783	1.00	reference	
	≥ 6	1181	0.99	0.85-1.15	0.847
Subjective sleep quality	0	332	1.00	reference	
	1	2029	0.86	0.68-1.10	0.233
	2	554	1.02	0.77-1.35	0.902
	3	49	0.67	0.35-1.30	0.233
Sleep latency	0	1399	1.00	reference	
	1	1038	0.82	0.69-0.97	0.022
	2	405	0.95	0.75-1.20	0.659
	3	122	1.02	0.69-1.50	0.920
Sleep duration	0	430	1.00	reference	
	1	994	1.15	0.90-1.45	0.262
	2	1444	0.94	0.75-1.18	0.579
	3	96	1.17	0.74-1.84	0.510
Habitual sleep efficiency	0	2030	1.00	reference	
	1	749	1.29	1.09-1.53	0.004
	2	118	0.804	0.53-1.21	0.296
	3	67	0.726	0.42-1.26	0.255
Sleep disturbance	0	956	1.00	reference	
	1	1946	0.97	0.83-1.14	0.725
	2	62	1.17	0.69-1.99	0.551
	3	0	-	-	-
Use of sleep medication	0	2644	1.00	reference	
	1	79	0.69	0.42-1.13	0.142
	2	54	0.68	0.37-1.24	0.202
	3	187	0.75	0.54-1.04	0.084
Daytime dysfunction	0	1417	1.00	reference	
	1	1234	0.96	0.82-1.13	0.626
	2	273	1.04	0.79-1.36	0.805

For peer review only - http://bmjopen.bmj.com/site/about/guidelines.xhtml

3	40	2.08	1.10-3.92	0.024

PSQI; Pittsburgh sleep quality index

Discussion

For peer review only

Lifestyle is an important cause of metabolic diseases, such as NAFLD. However, compared with nutrition or exercise, sleep disorder has gained less attention. Among the several sleep disorders, OSA and short sleep duration have been established as risk factors for NAFLD [6-13]. On the other hand, the detailed effects of sleep quality on the risk for NAFLD remain unclear. In the present study, we first evaluated the associations of NAFLD with all the elements of PSQI and found that the score for sleep medication use was significantly higher in patients without NAFLD than in those with NAFLD. In addition, the score for sleep medication use was associated with a relatively low odds ratio for NAFLD, whereas the score for daytime dysfunction was associated with a relatively high odds ratio for NAFLD. Our data revealed both the problems and the solutions for sleep disorder in NAFLD.

Previous studies have suggested the underlying mechanisms by which sleep quality contributes to NAFLD. Poor sleep quality activates the hypothalamic–pituitary–adrenal axis, thereby, enhancing the secretion of stress hormones, such as cortisol and

For peer review only - http://bmjopen.bmj.com/site/about/guidelines.xhtml

catecholamine, which can increase the risk for metabolic syndrome [14-16]. Other studies have shown the association between poor sleep quality and the hypothalamic orexin [17-18], which controls the secretion of the appetite-regulating hormones leptin and ghrelin [19]. These hormones play roles in the regulation of glucose metabolism [20]. In fact, poor sleep quality was reported to increase insulin resistance in patients with type 2 diabetes [21].

Although both short sleep duration and sleep quality have been associated with the disease severity and insulin resistance in NAFLD [9], the use of sleeping pills, regardless of the drug type, has been shown to improve sleep quality and extend sleeping time [22-25]. Therefore, the higher score for sleep medication use in non-NAFLD than in NAFLD implied the favorable effects of sleeping pills on metabolic factors. The results of this study suggested that sleeping pills may be a new treatment or preventive strategy for NAFLD in patients with sleep disorder. Indeed, the use of suvorexant, which targets the orexin system, for the treatment of insomnia has been shown to improve glycemic control in patients with type 2 diabetes mellitus [26]. Furthermore, ramelteon, which is a melatonin receptor agonist, may be beneficial for NAFLD by repairing the circadian rhythm, which is associated with glucose metabolism and insulin sensitivity [27].

Daytime dysfunction is a possible consequence of sleep disruption and may be

For peer review only - http://bmjopen.bmj.com/site/about/guidelines.xhtml

directly reflected by daytime sleepiness. This study supported the previous studies that reported the associations of daytime sleepiness with elevation of liver enzymes, insulin resistance, and the histologic features of NAFLD [9]. On the other hand, in this study, there was no difference in the mean daytime dysfunction score between the non-NAFLD and NAFLD groups. This result may have reflected the effects of possible OSA on NAFLD in some patients, based on the fact that daytime sleepiness is a major complaint of patients with OSA. In agreement with our results, the results in a previous study showed that the median Epworth sleepiness scale did not differ significantly between the non-NAFLD and NAFLD groups [9].

Although this study had the strength of a large sample size from multiple centers, it had some limitations. First, being a self-reported questionnaire, the PSQI assessment was subjective. Therefore, the associations between sleep quality and NAFLD need to be confirmed by objective methods, such as polysomnography and actigraphy. Second, this study was cross-sectional, and further longitudinal evaluation would be essential to reinforce the associations between sleep quality and NAFLD. Third, we were not able to identify the specific names of the sleeping medications. In the future, it would be interesting to know the sleeping medication with the most favorable effect on preventing NAFLD. Fourth, some of the results of this study on the association between sleep quality

For peer review only - http://bmjopen.bmj.com/site/about/guidelines.xhtml

and NAFLD were contrary to those in previous reports [8-10]; this may be explained by the fact that sleep quality could be affected by many factors, such as cultural, physical, psychological, and ethnic differences [28].

Conclusions

This study demonstrated that sleeping medication use and daytime dysfunction were associated with NAFLD. These findings suggested that improvement of daytime dysfunction and the use of sleeping pills could be the new goals and cutting edge treatment for NAFLD.

References

1. Zelber-Sagi S, Ratziu V, Oren R. Nutrition and physical activity in NAFLD: an overview of the epidemiological evidence. World J Gastroenterol. 2011; 17: 3377-89.
2. Angulo P, Kleiner DE, Dam-Larsen S, et al. Liver Fibrosis, but No Other Histologic Features, Is Associated With Long-term Outcomes of Patients With Nonalcoholic Fatty Liver Disease. Gastroenterology. 2015; 149: 389-97.
3. Musso G, Cassader M, Rosina F, et al. Impact of current treatments on liver disease, glucose metabolism and cardiovascular risk in non-alcoholic fatty liver disease

For peer review only - http://bmjopen.bmj.com/site/about/guidelines.xhtml

(NAFLD): a systematic review and meta-analysis of randomised trials. Diabetologia. 2012; 55: 885-904.

4. Vilar-Gomez E, Martinez-Perez Y, Calzadilla-Bertot L, et al. Weight Loss Through Lifestyle Modification Significantly Reduces Features of Nonalcoholic Steatohepatitis. Gastroenterology. 2015; 149: 367-78

5. Chalasani N, Younossi Z, Lavine JE, et al. The diagnosis and management of nonalcoholic fatty liver disease: Practice guidance from the American Association for the Study of Liver Diseases. Hepatology. 2018; 67: 328-57.

6. Aron-Wisnewsky J, Clement K, Pépin JL. Nonalcoholic fatty liver disease and obstructive sleep apnea. Metabolism. 2016; 65: 1124-35.

7. Imaizumi H, Takahashi A, Tanji N, et al. The Association between Sleep Duration and Non-Alcoholic Fatty Liver Disease among Japanese Men and Women.Obes Facts. 2015; 8: 234-42.

8. Kim CW, Yun KE, Jung HS et al. Sleep duration and quality in relation to non-alcoholic fatty liver disease in middle-aged workers and their spouses.J Hepatol. 2013; 59: 351-7.

9. Bernsmeier C, Weisskopf DM, Pflueger MO, et al. Sleep Disruption and Daytime Sleepiness Correlating with Disease Severity and Insulin Resistance in Non-

For peer review only

For peer review only - http://bmjopen.bmj.com/site/about/guidelines.xhtml

Alcoholic Fatty Liver Disease: A Comparison with Healthy Controls. PLoS One. 2015; 10: e0143293.

10. Marin-Alejandre BA, Abete I, Cantero I, et al. The Metabolic and Hepatic Impact of Two Personalized Dietary Strategies in Subjects with Obesity and Nonalcoholic Fatty Liver Disease: The Fatty Liver in Obesity (FLiO) Randomized Controlled Trial. Nutrients. 2019; 11: E2543.

11. Buysse DJ, Reynolds CF 3rd, Monk TH, et al. The Pittsburgh Sleep Quality Index: a new instrument for psychiatric practice and research. Psychiatry Res. 1989; 28: 193-213.

12. Wijarnpreecha K, Thongprayoon C, Panjawatanan P, et al. Short sleep duration and risk of nonalcoholic fatty liver disease: A systematic review and meta-analysis. J Gastroenterol Hepatol. 2016; 31: 1802-7.

13. Okamura T, Hashimoto Y, Hamaguchi M, et al. Short sleep duration is a risk of incident nonalcoholic fatty liver disease: a population-based longitudinal study. J Gastrointestin Liver Dis. 2019; 28: 73-81.

14. Cagampang FR, Poore KR, Hanson MA. Developmental origins of the metabolic syndrome: body clocks and stress responses. Brain Behav Immun. 2011; 25: 214-20.

15. Balbo M, Leproult R, Van Cauter E. Impact of sleep and its disturbances on

For peer review only

hypothalamo-pituitary-adrenal axis activity. Int J Endocrinol. 2010; 2010: 759234.

16. Spiegel K, Leproult R, Van Cauter E. Impact of sleep debt on metabolic and endocrine function. Lancet. 1999; 354: 1435-9.

17. Taheri S, Hafizi S. The orexins/hypocretins: hypothalamic peptides linked to sleep and appetite. Psychol Med. 2002; 32:955-8.

18. Sauchelli S, Jiménez-Murcia S, Fernández-García JC, et al. Interaction Between Orexin-A and Sleep Quality in Females in Extreme Weight Conditions. Eur Eat Disord Rev. 2016; 24: 510-7.

19. Littman AJ, Vitiello MV, Foster-Schubert K, et al. Sleep, ghrelin, leptin and changes in body weight during a 1-year moderate-intensity physical activity intervention. Int J Obes (Lond). 2007; 31: 466-75.

20. Tsuneki H, Wada T, Sasaoka T. Role of orexin in the regulation of glucose homeostasis. Acta Physiol (Oxf). 2010; 198: 335-48.

21. Yoda K, Inaba M, Hamamoto K, et al. Association between poor glycemic control, impaired sleep quality, and increased arterial thickening in type 2 diabetic patients. PLoS One. 2015; 10: e0122521.

22. Huedo-Medina TB, Kirsch I, Middlemass J, et al. Effectiveness of non-benzodiazepine hypnotics in treatment of adult insomnia: meta-analysis of data

For peer review only

For peer review only - http://bmjopen.bmj.com/site/about/guidelines.xhtml

submitted to the Food and Drug Administration. BMJ. 2012; 345: e8343.

23. Kuriyama A, Honda M, Hayashino Y. Ramelteon for the treatment of insomnia in adults: a systematic review and meta-analysis. Sleep Med. 2014; 15: 385-92.

24. Kanji S, Mera A, Hutton B, et al. Pharmacological interventions to improve sleep in hospitalised adults: a systematic review. BMJ Open. 2016; 6: e012108.

25. Kishi T, Matsunaga S, Iwata N. Suvorexant for Primary Insomnia: A Systematic Review and Meta-Analysis of Randomized Placebo-Controlled Trials. PLoS One. 2015; 10: e0136910.

26. Toi N, Inaba M, Kurajoh M, et al. Improvement of glycemic control by treatment for insomnia with suvorexant in type 2 diabetes mellitus. J Clin Transl Endocrinol. 2018; 15: 37-44.

27. Thomas AP, Hoang J, Vongbunyong K, et al. Administration of Melatonin and Metformin Prevents Deleterious Effects of Circadian Disruption and Obesity in Male Rats. Endocrinology. 2016; 157: 4720-4731.

28. Garfield V, Joshi R, Garcia-Hernandez J, et al. The relationship between sleep quality and all-cause, CVD and cancer mortality: the Southall and Brent REvisited study (SABRE). Sleep Med. 2019; 60: 230-5.

For peer review only

For peer review only - http://bmjopen.bmj.com/site/about/guidelines.xhtml

Footnotes

Contributors: AT, NT and HO contributed to the design of the present study. AT, MK,YA, MK, YK, and TO were responsible for data collection and overseeing the study procedures. The analysis was conducted by AT. The manuscript was written by AT, HO and YA, MK, MK, YK, TO, FM, MH, HI, KA, NT made contributions to the interpretation of the results in present study. All authors read and approved the final version of the manuscript.

Competing interests: The authors declare that they have no competing interests.

Patient consent: obtained

Ethics approval: The study protocol was approved by the ethics committee of Fukushima Medical University School of Medicine (#1636).

Data sharing statement: No additional data available

For peer review only - http://bmjopen.bmj.com/site/about/guidelines.xhtml

For peer review only

For peer review only - http://bmjopen.bmj.com/site/about/guidelines.xhtml

STROBE Statement—Checklist of items that should be included in reports of ***cohort studies***

	Item No	Recommendation	Page No
**Title and abstract**	1	(*a*) Indicate the study's design with a commonly used term in the title or the abstract	1
		(*b*) Provide in the abstract an informative and balanced summary of what was done and what was found	2
**Introduction**			
Background/rationale	2	Explain the scientific background and rationale for the investigation being reported	2-3
Objectives	3	State specific objectives, including any prespecified hypotheses	3
**Methods**			
Study design	4	Present key elements of study design early in the paper	5-7
Setting	5	Describe the setting, locations, and relevant dates, including periods of recruitment, exposure, follow-up, and data collection	5-7
Participants	6	(*a*) Give the eligibility criteria, and the sources and methods of selection of participants. Describe methods of follow-up	5
		(*b*) For matched studies, give matching criteria and number of exposed and unexposed	5
Variables	7	Clearly define all outcomes, exposures, predictors, potential confounders, and effect modifiers. Give diagnostic criteria, if applicable	5-7
Data sources/ measurement	8*	For each variable of interest, give sources of data and details of methods of assessment (measurement). Describe comparability of assessment methods if there is more than one group	5-7
Bias	9	Describe any efforts to address potential sources of bias	
Study size	10	Explain how the study size was arrived at	5
Quantitative variables	11	Explain how quantitative variables were handled in the analyses. If applicable, describe which groupings were chosen and why	
Statistical methods	12	(*a*) Describe all statistical methods, including those used to control for confounding	7
		(*b*) Describe any methods used to examine subgroups and interactions	7
		(*c*) Explain how missing data were addressed	
		(*d*) If applicable, explain how loss to follow-up was addressed	
		(*e*) Describe any sensitivity analyses	
**Results**			
Participants	13*	(a) Report numbers of individuals at each stage of study—eg numbers potentially eligible, examined for eligibility, confirmed eligible, included in the study, completing follow-up, and analysed	8
		(b) Give reasons for non-participation at each stage	
		(c) Consider use of a flow diagram	
Descriptive data	14*	(a) Give characteristics of study participants (eg demographic, clinical, social) and information on exposures and potential confounders	8-12
		(b) Indicate number of participants with missing data for each variable of interest	
		(c) Summarise follow-up time (eg, average and total amount)	
Outcome data	15*	Report numbers of outcome events or summary measures over time	8-12

For peer review only

For peer review only - http://bmjopen.bmj.com/site/about/guidelines.xhtml

Main results	16	(*a*) Give unadjusted estimates and, if applicable, confounder-adjusted estimates and their precision (eg, 95% confidence interval). Make clear which confounders were adjusted for and why they were included<br>(*b*) Report category boundaries when continuous variables were categorized<br>(*c*) If relevant, consider translating estimates of relative risk into absolute risk for a meaningful time period	8-12
Other analyses	17	Report other analyses done—eg analyses of subgroups and interactions, and sensitivity analyses	
**Discussion**			
Key results	18	Summarise key results with reference to study objectives	13
Limitations	19	Discuss limitations of the study, taking into account sources of potential bias or imprecision. Discuss both direction and magnitude of any potential bias	15
Interpretation	20	Give a cautious overall interpretation of results considering objectives, limitations, multiplicity of analyses, results from similar studies, and other relevant evidence	13-15
Generalisability	21	Discuss the generalisability (external validity) of the study results	
**Other information**			
Funding	22	Give the source of funding and the role of the funders for the present study and, if applicable, for the original study on which the present article is based	

*Give information separately for exposed and unexposed groups.

Note: An Explanation and Elaboration article discusses each checklist item and gives methodological background and published examples of transparent reporting. The STROBE checklist is best used in conjunction with this article (freely available on the Web sites of PLoS Medicine at http://www.plosmedicine.org/, Annals of Internal Medicine at http://www.annals.org/, and Epidemiology at http://www.epidem.com/). Information on the STROBE Initiative is available at http://www.strobe-statement.org.

For peer review only

For peer review only - http://bmjopen.bmj.com/site/about/guidelines.xhtml

BMJ Open

Effects of sleep quality on non-alcoholic fatty liver disease: A cross-sectional survey

Journal:	*BMJ Open*
Manuscript ID	bmjopen-2020-039947.R1
Article Type:	Original research
Date Submitted by the Author:	18-Aug-2020
Complete List of Authors:	Takahashi, Atsushi; Fukushima Medical University School of Medicine, Gastroenterology<br>Anzai, Yukio; Watari Hospital, Gastroenterology<br>Kuroda, Masahito ; Fukushima Red Cross Hospital, Gastroenterology<br>Kokubun, Masae ; Jusendo Hospital, total medical checkup<br>Kondo, Yuichiro ; Fujita General Hospital, Gastroenterology<br>Ogata, Takashi ; Masu Memorial Hospital, Gastroenterology<br>Fujita , Masashi; Fukushima Medical University School of Medicine, Gastroenterology<br>Hayashi, Manabu ; Fukushima Medical University School of Medicine, Gastroenterology<br>Imaizumi, Hiromichi ; Fukushima Medical University School of Medicine, Gastroenterology<br>Abe, Kazumichi ; Fukushima Medical University School of Medicine, Gastroenterology<br>Tanji, Nobuo; Watari Hospital, Gastroenterology<br>Ohira, Hiromasa; Fukushima Medical University School of Medicine, Gastroenterology
**Primary Subject Heading**:	Gastroenterology and hepatology
Secondary Subject Heading:	Gastroenterology and hepatology
Keywords:	Gastroenterology < INTERNAL MEDICINE, Hepatobiliary disease < GASTROENTEROLOGY, SLEEP MEDICINE

SCHOLARONE™
Manuscripts

I, the Submitting Author has the right to grant and does grant on behalf of all authors of the Work (as defined in the below author licence), an exclusive licence and/or a non-exclusive licence for contributions from authors who are: i) UK Crown employees; ii) where BMJ has agreed a CC-BY licence shall apply, and/or iii) in accordance with the terms applicable for US Federal Government officers or employees acting as part of their official duties; on a worldwide, perpetual, irrevocable, royalty-free basis to BMJ Publishing Group Ltd ("BMJ") its licensees and where the relevant Journal is co-owned by BMJ to the co-owners of the Journal, to publish the Work in this journal and any other BMJ products and to exploit all rights, as set out in our licence.

The Submitting Author accepts and understands that any supply made under these terms is made by BMJ to the Submitting Author unless you are acting as an employee on behalf of your employer or a postgraduate student of an affiliated institution which is paying any applicable article publishing charge ("APC") for Open Access articles. Where the Submitting Author wishes to make the Work available on an Open Access basis (and intends to pay the relevant APC), the terms of reuse of such Open Access shall be governed by a Creative Commons licence – details of these licences and which Creative Commons licence will apply to this Work are set out in our licence referred to above.

Other than as permitted in any relevant BMJ Author's Self Archiving Policies, I confirm this Work has not been accepted for publication elsewhere, is not being considered for publication elsewhere and does not duplicate material already published. I confirm all authors consent to publication of this Work and authorise the granting of this licence.

For peer review only

Effects of sleep quality on non-alcoholic fatty liver disease: A cross-sectional survey

Atsushi Takahashi[1], Yukio Anzai[2], Masahito Kuroda[3], Masae Kokubun[4], Yuichiro Kondo[5], Takashi Ogata[6], Fujita Masashi[1], Manabu Hayashi[1], Hiromichi Imaizumi[1], Kazumichi Abe[1], Nobuo Tanji[2], Hiromasa Ohira[1]

[1]Department of Gastroenterology, Fukushima Medical University School of Medicine, Fukushima, Japan, [2]Department of Gastroenterology, Watari Hospital, Fukushima, Japan, [3]Department of Gastroenterology, Fukushima Red Cross Hospital, Fukushima, Japan, [4]Institution for Total Medical Checkup, Jusendo Clinic, Koriyama, Japan, [5]Department of Gastroenterology, Fujita General Hospital, Date-gun, Japan, [6]Department of Gastroenterology, Masu Memorial Hospital, Nihonmatsu, Japan

Correspondence to: Atsushi Takahashi, M.D.

Department of Gastroenterology, Fukushima Medical University, 1 Hikarigaoka, Fukushima 960-1295, Japan

E-mail: junior@fmu.ac.jp

Telephone: +81-24-547-1202; Fax: +81-24-547-2005

For peer review only

For peer review only - http://bmjopen.bmj.com/site/about/guidelines.xhtml

Word count: 2446

For peer review only

For peer review only - http://bmjopen.bmj.com/site/about/guidelines.xhtml

Abstract

Background The effects of sleep quality on the risk of developing non-alcoholic fatty liver disease (NAFLD) remain uncertain. The purpose of this study was to clarify the association between sleep quality and NAFLD.

Methods The data of 4,828 participants who underwent health check-ups at 4 hospitals were analyzed. Sleep quality was evaluated by the Pittsburgh Sleep Quality Index (PSQI), which comprised 7 elements scored from 0 to 3. The global PSQI score and the score for each element were compared between NAFLD and non-NAFLD groups separately by sex. Logistic regression analysis was performed to determine the association between NAFLD and each PSQI score.

Results In both men and women, the mean PSQI score for sleep medication use was significantly higher in non-NAFLD than in NAFLD. With regard to sleep medication use in men, the odds ratio (OR) (95% confidence interval (CI)) for NAFLD was lower with a score of 3 (OR 0.60, 95% CI 0.38–0.95) than with a score of 0 on multivariate logistic regression analysis adjusted for age, smoking habits, and physical activity. The odds ratio for NAFLD based on daytime dysfunction was also higher with a score of 3 than with a score of 0 in both men (OR 2.82, 95% CI 1.39–5.75) and women (OR 2. 08, 95% CI 1.10–3.92). After adjustment for body mass index, the sleep latency scores in

For peer review only

men and daytime dysfunction in women were associated with NAFLD.

Conclusion Sleep quality was associated with NAFLD, and there were sex differences.

Key words: sleep quality, non-alcoholic fatty liver disease, sleep medication, daytime dysfunction, sleep latency

Strengths and limitations of this study

- The strength of this study lies in the large sample size from multiple centers.
- This is the first study to use all 7 element scores of the Pittsburgh Sleep Quality Index to assess the association between sleep quality and non-alcoholic fatty liver disease.
- The present study was based on a self-reported questionnaire (Pittsburgh Sleep Quality Index); therefore, the associations between sleep quality and NAFLD need to be confirmed by objective methods.

For peer review only

Introduction

Non-alcoholic fatty liver disease (NAFLD) is the most common chronic liver disease, probably because of the widespread prevalence of unhealthy lifestyle habits, such as excess nutritional intake or less physical activity [1]. In patients with NAFLD, liver fibrosis has been associated with liver-related outcomes and mortality [2]. Lifestyle interventions, such as diet modification and exercise to achieve weight loss, are fundamental in the treatment of patients with NAFLD; in fact, weight loss of more than 7% to 10% has been reported to improve liver fibrosis in NAFLD patients [3-5]. However, compared with nutrition or exercise, sleep disorder has attracted less attention. Of the several sleep disorders, obstructive sleep apnea (OSA) and short sleep duration have been established as risk factors for NAFLD [6-11]. On the other hand, only a few reports of the association between sleep quality and NAFLD have been published [9, 12-13].

The Pittsburgh Sleep Quality Index (PSQI) is an established and widely used method for sleep quality evaluation; it comprises 7 elements, including subjective sleep quality, sleep latency, sleep duration, habitual sleep efficiency, sleep disturbance, sleep medication use, and daytime dysfunction [14]. In previous reports of the sleep quality of patients with NAFLD, only the results of the global PSQI score or some components of

For peer review only

For peer review only - http://bmjopen.bmj.com/site/about/guidelines.xhtml

the PSQI were assessed [9, 12-13]. Therefore, the associations of all components of the PSQI with NAFLD are poorly understood. In addition to sleep time distribution [8], the impact of sleep quality on NAFLD differs by sex [9]. In the present study, the aim was to clarify the association between sleep quality and NAFLD by analyzing all elements of the PSQI separately by sex and by adjusting for confounding factors.

Methods

Subjects

The subjects of this study were 6,138 Japanese men and women who underwent health check-ups from May 2013 to March 2014 at the Watari Hospital Health Center, Jusendo Hospital, Fujita General Hospital, Fukushima Red Cross Hospital, and Masu Memorial Hospital in Fukushima Prefecture, Japan. A total of 423 subjects who had insufficient data and 887 subjects who had chronic liver disease secondary to other causes (i.e., hepatitis C antibody-positive, hepatitis B surface antigen-positive, and alcoholic consumption >30 g/day for men and >20 g/day for women) were excluded. NAFLD was defined as fatty liver detected by ultrasound in the absence of the above-mentioned causes of chronic liver disease based on an established guideline [15]. Accordingly, 4,828 subjects (1,864 men and 2,964 women) were eligible for this study. The study

For peer review only - http://bmjopen.bmj.com/site/about/guidelines.xhtml

protocol was approved by the ethics committee of Fukushima Medical University School of Medicine (#1636). All patients provided written, informed consent prior to study participation, and this study was conducted in conformity with the ethical guidelines of the Declaration of Helsinki.

Measurements

Before the examination, the subjects were asked to complete a questionnaire regarding their ordinary sleep using the PSQI scale and lifestyle factors, including smoking habits, drinking habits, and physical activity. The PSQI scale included 19 self-administered questions that generated 7 elements, including subjective sleep quality, sleep latency, sleep duration, habitual sleep efficiency, sleep disturbance, sleep medication use, and daytime dysfunction. The score for each element ranged from 0 to 3, as follows: 1) subjective sleep quality (0 = very good, 1 = fairly good, 2 = fairly bad, and 3 = very bad; 2) sleep latency, which was defined as the sum of the scores of a) the required time in minutes to fall asleep each night (0 for ≤15 minutes, 1 for 16-30 minutes, 2 for 31-60 minutes, 3 for > 60 minutes) and b) the frequency of falling asleep within 30 minutes (0 for not during the past month, 1 for less than once a week, 2 for once or twice a week, 3 for three or more times a week), assigning a score of 0 for a sum of 0, 1 for a sum of 1-2,

For peer review only - http://bmjopen.bmj.com/site/about/guidelines.xhtml

For peer review only

2 for a sum of 3-4, and 3 for a sum of 5-6; 3) sleep duration (0 for >7 hours, 1 for 6-≤7 hours, 2 for 5-<6 hours, and 3 for <5 hours); 4) habitual sleep efficiency, which was defined as the proportion of hours slept to hours spent in bed (0 for ≥85%, 1 for 75% to 84%, 2 for 65% to 74%, and 3 for <65%); 5) frequency of sleep disturbance, which was defined as having trouble sleeping for some reason (0 for not during the past month, 1 for less than once a week, 2 for once or twice a week, 3 for three or more times a week), assigning 0 for a total score of 0, 1 for a total score of 1-9, 2 for a total score of 10-18, and 3 for a total score of 19-27; 6) sleep medication use (0 for no use during the past month, 1 for less than once a week, 2 for once or twice a week, and 3 for three or more times a week; and 7) frequency of daytime dysfunction, which was defined as the sum of the scores of a) having trouble staying awake while driving, eating meals, or engaging in social activity (0 for never, 1 for once or twice, 2 for once or twice each week, 3 for three or more times each week) and b) having trouble in sustaining enough enthusiasm to get things done (0 for no problem at all, 1 for only a very slight problem, 2 for somewhat of a problem, 3 for a serious problem), assigning 0 for a total score of 0, 1 for a total score of 1-2, 2 for a total score of 3-4, and 3 for a total score of 5-6. The cumulative score of these elements was defined as the global score, which ranged from 0 to 21. Poor sleepers were identified based on a global score of >5.

For peer review only

For peer review only - http://bmjopen.bmj.com/site/about/guidelines.xhtml

Physical activity was defined as walking or an equivalent amount of physical activity for more than 1 hour a day. The subjects were examined after an overnight fast. Ultrasound was used to evaluate hepatic steatosis. An ultrasound diagnosis of fatty liver was defined as a bright liver, with increased liver echogenicity compared with that in the kidneys, vascular blurring, and deep attenuation of the liver [16].

Statistical analysis

All statistical analyses were conducted separately by sex, based on the fact that both sleep status and NAFLD have sex differences. The two groups (men and women) were compared using the Mann-Whitney U-test and the χ^2 test for categorical variables. For continuous variables, analysis of covariance was performed to determine any significant differences between the non-NAFLD and NAFLD groups, after adjustments for age, smoking habits, and physical exercise. After adjusting for the possible confounding factors, such as age, body mass index (BMI), smoking habits, and physical activity, logistic regression analysis was conducted to assess the relationships of sleep disorder, based on the global PSQI score and the other 7 elements, with NAFLD. The values for odds ratios (ORs) with 95% confidence intervals (CIs) were calculated from the logistic regression analysis. Statistical analyses were performed using SPSS 25.0 for Windows

For peer review only

For peer review only - http://bmjopen.bmj.com/site/about/guidelines.xhtml

(SPSS, Inc., Chicago, IL, USA). A *p* value of <0.05 was considered significant.

Patient and Public involvement

Patients and public were not included in the present study. Patient consent is not required.

Results

Characteristics of the participants

The participants' characteristics were compared by sex to clarify any sex differences (Table 1). The mean age was significantly higher in women than in men (55.5 years vs. 56.6 years; p = 0.002). Compared with men, women had significantly lower BMI and sleep duration; lower proportions of subjects with a smoking history and NAFLD; higher level of physical activity; and significantly higher global PSQI scores (p <0.001 for all).

Table 1 Characteristics of participants

	Men (n=1864)	Women (n=2964)	*p* value
Age, y	55.5 ± 12.7	56.6 ± 12.4	0.002
Body mass index, kg/m^2	23.4 ±3.3	22.9 ± 3.5	< 0.001
Sleep duration min/day	388 ± 63	380 ± 59	< 0.001
Physical activity	41.5% (774)	47.1% (1395)	< 0.001

For peer review only - http://bmjopen.bmj.com/site/about/guidelines.xhtml

Current smoker	25.1% (467)	5.6% (165)	< 0.001
NAFLD	47.0% (876)	37.1% (1099)	< 0.001
ALT, U/L	24.5 ± 15.5	20.7 ± 15.2	< 0.001
Global PSQI score	4.9 ± 2.6	5.2 ± 2.6	< 0.001

NAFLD: non-alcoholic fatty liver disease, *ALT:* alanine aminotransferase, *PSQI:* Pittsburgh sleep quality index

Comparison between non-NAFLD and NAFLD

The mean score for sleep medication use was significantly higher in those without NAFLD than in those with NAFLD in both men (0.21 ± 0.74 vs. 0.13 ± 0.59; $p = 0.044$) and women (0.28 ± 0.82 vs. 0.20 ± 0.70; $p = 0.030$) (Table 2). In men, the mean scores for sleep latency (0.58 ± 0.74 vs. 0.63 ± 0.78; $p = 0.066$) and daytime dysfunction (0.62 ± 0.72 vs. 0.71 ± 0.80; $p = 0.074$) tended to be lower in those without NAFLD than in those with NAFLD.

Table 2 Comparison of each PSQI score between the groups with and without NAFLD

	With NAFLD	Without NAFLD	*p* value
Men			
Global PSQI score	5.02 ± 2.65	4.85 ± 2.47	0.216
Subjective sleep quality	1.14 ± 0.61	1.07 ± 0.59	0.077
Sleep latency	0.63 ± 0.78	0.58 ± 0.74	0.066
Sleep duration	1.31 ± 0.81	1.27 ± 0.82	0.863
Habitual sleep efficiency	0.40 ± 0.66	0.40 ± 0.65	0.808
Sleep disturbance	0.70 ± 0.51	0.69 ± 0.50	0.414
Use of sleep medication	0.13 ± 0.59	0.21 ± 0.74	0.044
Daytime dysfunction	0.71 ± 0.80	0.62 ± 0.72	0.074
Women			
Global PSQI score	5.19 ± 2.59	5.29 ± 2.64	0.409

For peer review only - http://bmjopen.bmj.com/site/about/guidelines.xhtml

Subjective sleep quality	1.11 ± 0.60	1.11 ± 0.59	0.935
Sleep latency	0.72 ± 0.85	0.76 ± 0.84	0.443
Sleep duration	1.40 ± 0.77	1.41 ± 0.78	0.314
Habitual sleep efficiency	0.40 ± 0.63	0.40 ± 0.70	0.672
Sleep disturbance	0.69 ± 0.51	0.70 ± 0.50	0.947
Use of sleep medication	0.20 ± 0.70	0.28 ± 0.82	0.030
Daytime dysfunction	0.66 ± 0.73	0.63 ± 0.69	0.390

PSQI: Pittsburgh sleep quality index

Association between sleep disorder and NAFLD

In men, the odds ratio for NAFLD was lower with a score of 3 for sleep medication use (OR 0.60, 95%CI 0.38-0.95, p=0.030) than with a score of 0 (no sleep medication use) (Table 3); in women, the tendency was similar, but the odds ratio for NAFLD was not significant (OR 0.75, 95%CI 0.54-1.04, p=0.084) (Table 4). After further adjustment for BMI, sleep medication use was not associated with NAFLD in both men and women.

The odds ratio for NAFLD was higher with a daytime dysfunction score of 3 than a score of 0 in both men (OR 2.82, 95%CI 1.39-5.75, p=0.004) and women (OR 2.08, 95%CI 1.10-3.92, p=0.024). After further adjustments for BMI, daytime dysfunction was not associated with NAFLD in men, but it was in women with a daytime dysfunction score of 3 compared to those with a score of 0 (OR 2.62, 95%CI 1.20-5.72, p=0.015).

In men, the odds ratio for NAFLD was higher with a score of 2 for sleep latency (OR

For peer review only - http://bmjopen.bmj.com/site/about/guidelines.xhtml

1.66, 95%CI 1.14-2.02, *p*=0.008) than with a score of 0 after adjustment for BMI. In women, the odds ratio for NAFLD was lower with a score of 1 for sleep latency (OR 0.82, 95%CI 0.69-0.97, *p*=0.022) than with a score of 0. Moreover, the odds ratio for NAFLD was higher with a score of 1 for habitual sleep efficiency (OR 1.29, 95%CI 1.09-1.53, *p*=0.004) than with a score of 0 in women. After further adjustments for BMI, sleep latency and habitual sleep efficiency were not associated with NAFLD in women.

The present results showed no associations of NAFLD with the global PSQI score, subjective sleep quality score, sleep duration score, and sleep disturbance score.

Table 3 Odds ratio of NAFLD by the PSQI and its components in men (n=1864)

	Score	N	Model 1		Model 2	
			OR (95% CI)	*p*	OR (95% CI)	*p*
Global PSQI score	≤ 5	1202	1.00 (reference)		1.00 (reference)	
	≥ 6	662	1.08 (0.89-1.30)	0.458	1.17 (0.94-1.47)	0.161
Subjective sleep quality	0	223	1.00 (reference)		1.00 (reference)	
	1	1252	1.10 (0.83-1.47)	0.506	1.17 (0.83-1.64)	0.377
	2	363	1.24 (0.89-1.75	0.207	1.28 (0.86-1.90)	0.224
	3	26	2.02 (0.87-4.67)	0.101	1.68 (0.64-4.45)	0.295
Sleep latency	0	1011	1.00 (reference)		1.00 (reference)	
	1	624	1.05 (0.86-1.29)	0.622	1.03 (0.82-1.31)	0.781
	2	186	1.37 (1.00-1.88)	0.050	1.66 (1.14-2.02)	0.008
	3	43	1.31 (0.70-2.43)	0.398	1.31 (0.63-2.74)	0.467
Sleep duration	0	368	1.00 (reference)		1.00 (reference)	
	1	656	0.99 (0.76-1.28)	0.923	0.91 (0.67-1.23)	0.537
	2	778	1.01 (0.78-1.31)	0.937	0.89 (0.66-1.21)	0.458

For peer review only - http://bmjopen.bmj.com/site/about/guidelines.xhtml

For peer review only

	3	62	1.04 (0.60-1.80)	0.894	1.00 (0.52-1.93)	0.999
Habitual sleep efficiency	0	1262	1.00 (reference)		1.00 (reference)	
	1	500	1.00 (0.81-1.23)	0.991	1.01 (0.79-1.29)	0.931
	2	64	0.94 (0.57-1.57)	0.820	0.79 (0.42-1.47)	0.456
	3	38	1.23 (0.64-2.37)	0.534	1.71 (0.82-3.55)	0.152
Sleep disturbance	0	607	1.00 (reference)		1.00 (reference)	
	1	1218	1.07 (0.88-1.30)	0.499	1.08 (0.86-1.37)	0.493
	2	38	1.13 (0.58-2.19)	0.726	0.93 (0.41-2.10)	0.852
	3	1	-	-	-	-
Use of sleep medication	0	1730	1.00 (reference)		1.00 (reference)	
	1	26	0.97 (0.44-2.12)	0.939	1.09 (0.46-2.62)	0.842
	2	19	1.06 (0.43-2.65)	0.894	1.40 (0.53-3.90)	0.522
	3	89	0.60 (0.38-0.95)	0.030	0.61 (0.36-1.04)	0.068
Daytime dysfunction	0	912	1.00 (reference)		1.00 (reference)	
	1	702	1.00 (0.82-1.22)	0.992	1.16 (0.92-1.47)	0.203
	2	210	1.11 (0.82-1.50)	0.520	1.16 (0.81-1.66)	0.414
	3	40	2.82 (1.39-5.75)	0.004	2.04 (0.92-4.54)	0.079

Model 1: adjustment for age, smoking habits, and physical activity; Model 2: Model 1 plus adjustment for body mass index. *PSQI:* Pittsburgh sleep quality index

Table 4 Odds ratio of NAFLD by the PSQI and its components in women (n=2964)

	Score	N	Model 1		Model 2	
			OR (95% CI)	*p*	OR (95% CI)	*p*
Global PSQI score	≤ 5	1783	1.00 (reference)		1.00 (reference)	
	≥ 6	1181	0.99 (0.85-1.15)	0.847	1.17 (0.94-1.47)	0.161
Subjective sleep quality	0	332	1.00 (reference)		1.00 (reference)	
	1	2029	0.86 (0.68-1.10)	0.233	0.93 (0.70-1.23)	0.610
	2	554	1.02 (0.77-1.35)	0.902	1.11 (0.80-1.55)	0.538
	3	49	0.67 (0.35-1.30)	0.233	0.81 (0.39-1.68)	0.565
Sleep latency	0	1399	1.00 (reference)		1.00 (reference)	
	1	1038	0.82 (0.69-0.97)	0.022	0.83 (0.68-1.01)	0.068
	2	405	0.95 (0.75-1.20)	0.659	0.98 (0.74-1.29)	0.865
	3	122	1.02 (0.69-1.50)	0.920	0.82 (0.52-1.29)	0.392
Sleep duration	0	430	1.00 (reference)		1.00 (reference)	
	1	994	1.15 (0.90-1.45)	0.262	1.22 (0.92-1.60)	0.169

For peer review only

	2	1444	0.94 (0.75-1.18)	0.579	0.92 (0.70-1.20)	0.520
	3	96	1.17 (0.74-1.84)	0.510	1.07 (0.63-1.82)	0.810
Habitual sleep efficiency	0	2030	1.00 (reference)		1.00 (reference)	
	1	749	1.29 (1.09-1.53)	0.004	1.21 (0.99-1.48)	0.069
	2	118	0.804 (0.53-1.21)	0.296	0.96 (0.60-1.54)	0.859
	3	67	0.726 (0.42-1.26)	0.255	0.66 (0.37-1.25)	0.199
Sleep disturbance	0	956	1.00 (reference)		1.00 (reference)	
	1	1946	0.97 (0.83-1.14)	0.725	0.97 (0.80-1.17)	0.763
	2	62	1.17 (0.69-1.99)	0.551	1.30 (0.70-2.44)	0.410
	3	0	-			-
Use of sleep medication	0	2644	1.00 (reference)		1.00 (reference)	
	1	79	0.69 (0.42-1.13)	0.142	0.97 (0.55-1.72)	0.919
	2	54	0.68 (0.37-1.24)	0.202	0.62 (0.31-1.22)	0.166
	3	187	0.75 (0.54-1.04)	0.084	0.69 (0.46-1.02)	0.061
Daytime dysfunction	0	1417	1.00 (reference)		1.00 (reference)	
	1	1234	0.96 (0.82-1.13)	0.626	0.98 (0.81-1.19)	0.848
	2	273	1.04 (0.79-1.36)	0.805	0.94 (0.68-1.30)	0.718
	3	40	2.08 (1.10-3.92)	0.024	2.62 (1.20-5.72)	0.015

Model 1: adjustment for age, smoking habits, and physical activity; Model 2: Model 1 plus adjustment for body mass index. *PSQI:* Pittsburgh sleep quality index

Discussion

In the present study, the associations of NAFLD with all the elements of the PSQI were first evaluated, and it was found that the score for sleep medication use was significantly higher in patients without NAFLD than in those with NAFLD. In addition, the score for sleep medication use in men was associated with a relatively low odds ratio for NAFLD, whereas the score for daytime dysfunction was associated with a

For peer review only - http://bmjopen.bmj.com/site/about/guidelines.xhtml

relatively high odds ratio for NAFLD in both sexes. The scores for sleep latency in both sexes and habitual sleep efficiency in women were also associated with NAFLD.

Previous studies have suggested the underlying mechanisms by which sleep quality contributes to NAFLD. Poor sleep quality activates the hypothalamic–pituitary–adrenal (HPA) axis, thereby enhancing the secretion of stress hormones, such as cortisol and catecholamines, which can increase the risk for metabolic syndrome [17-19]. Other studies have shown the association between poor sleep quality and hypothalamic orexin [20-21], which controls the secretion of the appetite-regulating hormones leptin and ghrelin [22]. These hormones play roles in the regulation of glucose metabolism [23]. In fact, poor sleep quality was reported to increase insulin resistance in patients with type 2 diabetes mellitus [24]. Estrogen activates the HPA axis response to stress [25], and insulin resistance due to poor sleep quality develops only in women [26], explaining possible sex differences in the relationship between sleep quality and NAFLD.

Although both short sleep duration and sleep quality have been associated with disease severity and insulin resistance in NAFLD [12], the use of sleeping pills, regardless of the drug type, has been shown to improve sleep quality and extend sleeping time [27-31]. On other hand, ramelteon, which is a melatonin receptor agonist, may be beneficial for NAFLD by repairing the circadian rhythm, which is associated with

For peer review only - http://bmjopen.bmj.com/site/about/guidelines.xhtml

glucose metabolism and insulin sensitivity [32]. The effect of sleeping pills for preventing NAFLD may be worth examining.

Daytime dysfunction is a possible consequence of sleep disruption and may be directly reflected by daytime sleepiness. The present study supported the previous studies that reported the associations of daytime sleepiness with elevations of liver enzymes, insulin resistance, and the histologic features of NAFLD [12]. On the other hand, in the present study, there was no difference in the mean daytime dysfunction score between the non-NAFLD and NAFLD groups. In agreement with the present results, the results of a previous study showed that the median Epworth sleepiness scale did not differ significantly between the non-NAFLD and NAFLD groups [12].

Obesity is a major risk factor for NAFLD. Therefore, scores of the PSQI except daytime dysfunction in women were not associated with NAFLD after adjustment for BMI. This suggests that the effects of sleep quality on NAFLD are mediated by obesity because of the strong association between obesity and sleep quality [33]. On the other hand, the prevalence of NAFLD is much higher than would be expected from a population with similar BMI in the present study. This may be explained by the fact that subjects in the present study often had NAFLD with normal or low body weight, and ultrasound has a high sensitivity for detection of fatty liver in such persons. The high

For peer review only - http://bmjopen.bmj.com/site/about/guidelines.xhtml

proportion of lean NAFLD in Japan compared to western countries may support the above interpretation [34].

Although this study had the strength of a large sample size from multiple centers, it had some limitations. First, being a self-reported questionnaire, the PSQI assessment was subjective. Therefore, the associations between sleep quality and NAFLD need to be confirmed by objective methods, such as polysomnography and actigraphy. Second, this study was cross-sectional and evaluated sleep characteristics at the same time as NAFLD. Therefore, further longitudinal evaluation would be essential to elucidate the causal relationship between sleep quality and NAFLD. Third, the specific names of the sleep medications being taken by the patients were not identified. In the future, it would be interesting to know which sleep medication has the most favorable effect on preventing NAFLD. Fourth, some of the results of this study on the association between sleep quality and NAFLD were contrary to those in previous reports [9, 12-13]. This may be explained by the fact that sleep quality could be affected by many factors, such as cultural, physical, psychological, and ethnic differences [35]. Fifth, the degree of fatty liver was not evaluated by semi-quantitative ultrasonographic indices in the present study. Future studies should explore the association between sleep quality and the degree of fat or fibrosis in NAFLD.

For peer review only - http://bmjopen.bmj.com/site/about/guidelines.xhtml

Conclusions

This study demonstrated that sleep medication use and daytime dysfunction were associated with NAFLD. These findings suggest that additional studies are needed to assess the importance, if any, of sleeping pills for the treatment of NAFLD.

Footnotes

Contributors: AT, NT, and HO contributed to the design of the present study. AT, MK, YA, MK, YK, and TO were responsible for data collection and overseeing the study procedures. The analysis was conducted by AT. The manuscript was written by AT and HO, and YA, MK, MK, YK, TO, FM, MH, HI, KA, and NT made contributions to the interpretation of the results in present study. All authors read and approved the final version of the manuscript.

Competing interests: The authors declare that they have no competing interests.

Funding: There are no funders to report for this submission.

For peer review only - http://bmjopen.bmj.com/site/about/guidelines.xhtml

Patient consent: obtained

Ethics approval: The study protocol was approved by the ethics committee of Fukushima Medical University School of Medicine (#1636).

Data sharing statement: No additional data available

References

1. Zelber-Sagi S, Ratziu V, Oren R. Nutrition and physical activity in NAFLD: an overview of the epidemiological evidence. World J Gastroenterol. 2011; 17: 3377-89.

2. Angulo P, Kleiner DE, Dam-Larsen S, et al. Liver Fibrosis, but No Other Histologic Features, Is Associated With Long-term Outcomes of Patients With Nonalcoholic Fatty Liver Disease. Gastroenterology. 2015; 149: 389-97.

3. Musso G, Cassader M, Rosina F, et al. Impact of current treatments on liver disease, glucose metabolism and cardiovascular risk in non-alcoholic fatty liver disease (NAFLD): a systematic review and meta-analysis of randomised trials.

For peer review only - http://bmjopen.bmj.com/site/about/guidelines.xhtml

Diabetologia. 2012; 55: 885-904.

4. Vilar-Gomez E, Martinez-Perez Y, Calzadilla-Bertot L, et al. Weight Loss Through Lifestyle Modification Significantly Reduces Features of Nonalcoholic Steatohepatitis. Gastroenterology. 2015; 149: 367-78
5. Chalasani N, Younossi Z, Lavine JE, et al. The diagnosis and management of nonalcoholic fatty liver disease: Practice guidance from the American Association for the Study of Liver Diseases. Hepatology. 2018; 67: 328-57.
6. Aron-Wisnewsky J, Clement K, Pépin JL. Nonalcoholic fatty liver disease and obstructive sleep apnea. Metabolism. 2016; 65: 1124-35.
7. Umbro I, Fabiani V, Fabiani M, et al. Association between non-alcoholic fatty liver disease and obstructive sleep apnea. World J Gastroenterol. 2020; 26:2669-81.
8. Imaizumi H, Takahashi A, Tanji N, et al. The Association between Sleep Duration and Non-Alcoholic Fatty Liver Disease among Japanese Men and Women.Obes Facts. 2015; 8: 234-42.
9. Kim CW, Yun KE, Jung HS et al. Sleep duration and quality in relation to non-alcoholic fatty liver disease in middle-aged workers and their spouses.J Hepatol. 2013; 59: 351-7.
10. Wijarnpreecha K, Thongprayoon C, Panjawatanan P, et al. Short sleep duration and

For peer review only - http://bmjopen.bmj.com/site/about/guidelines.xhtml

For peer review only

risk of nonalcoholic fatty liver disease: A systematic review and meta-analysis. J Gastroenterol Hepatol. 2016; 31: 1802-7.

11. Okamura T, Hashimoto Y, Hamaguchi M, et al. Short sleep duration is a risk of incident nonalcoholic fatty liver disease: a population-based longitudinal study. J Gastrointestin Liver Dis. 2019; 28: 73-81.
12. Bernsmeier C, Weisskopf DM, Pflueger MO, et al. Sleep Disruption and Daytime Sleepiness Correlating with Disease Severity and Insulin Resistance in Non-Alcoholic Fatty Liver Disease: A Comparison with Healthy Controls. PLoS One. 2015; 10: e0143293.
13. Marin-Alejandre BA, Abete I, Cantero I, et al. The Metabolic and Hepatic Impact of Two Personalized Dietary Strategies in Subjects with Obesity and Nonalcoholic Fatty Liver Disease: The Fatty Liver in Obesity (FLiO) Randomized Controlled Trial. Nutrients. 2019; 11: E2543.
14. Buysse DJ, Reynolds CF 3rd, Monk TH, et al. The Pittsburgh Sleep Quality Index: a new instrument for psychiatric practice and research. Psychiatry Res. 1989; 28: 193-213.
15. Wong VW, Chan WK, Chitturi S, et al. Asia-Pacific Working Party on Non-alcoholic Fatty Liver Disease guidelines 2017-Part 1: Definition, risk factors

For peer review only - http://bmjopen.bmj.com/site/about/guidelines.xhtml

and assessment. J Gastroenterol Hepatol. 2018; 33:70-85.

16. Farrell GC, Chitturi S, Lau GK, Sollano JD. Guidelines for the assessment and management of non-alcoholic fatty liver disease in the Asia-Pacific region: executive summary. J Gastroenterol Hepatol. 2007; 22:775–7.
17. Cagampang FR, Poore KR, Hanson MA. Developmental origins of the metabolic syndrome: body clocks and stress responses. Brain Behav Immun. 2011; 25: 214-20.
18. Balbo M, Leproult R, Van Cauter E. Impact of sleep and its disturbances on hypothalamo-pituitary-adrenal axis activity. Int J Endocrinol. 2010; 2010: 759234.
19. Spiegel K, Leproult R, Van Cauter E. Impact of sleep debt on metabolic and endocrine function. Lancet. 1999; 354: 1435-9.
20. Taheri S, Hafizi S. The orexins/hypocretins: hypothalamic peptides linked to sleep and appetite. Psychol Med. 2002; 32:955-8.
21. Sauchelli S, Jiménez-Murcia S, Fernández-García JC, et al. Interaction Between Orexin-A and Sleep Quality in Females in Extreme Weight Conditions. Eur Eat Disord Rev. 2016; 24: 510-7.
22. Littman AJ, Vitiello MV, Foster-Schubert K, et al. Sleep, ghrelin, leptin and changes in body weight during a 1-year moderate-intensity physical activity intervention. Int J Obes (Lond). 2007; 31: 466-75.

For peer review only

For peer review only - http://bmjopen.bmj.com/site/about/guidelines.xhtml

23. Tsuneki H, Wada T, Sasaoka T. Role of orexin in the regulation of glucose homeostasis. Acta Physiol (Oxf). 2010; 198: 335-48.

24. Yoda K, Inaba M, Hamamoto K, et al. Association between poor glycemic control, impaired sleep quality, and increased arterial thickening in type 2 diabetic patients. PLoS One. 2015; 10: e0122521.

25. Da Silva JA. Sex hormones and glucocorticoids: interactions with the immune system. Ann N Y Acad Sci. 1999; 876:102-17

26. Suarez EC. Brain Behav Immun. Self-reported symptoms of sleep disturbance and inflammation, coagulation, insulin resistance and psychosocial distress: evidence for gender disparity. 2008; 22:960-8.

27. Huedo-Medina TB, Kirsch I, Middlemass J, et al. Effectiveness of non-benzodiazepine hypnotics in treatment of adult insomnia: meta-analysis of data submitted to the Food and Drug Administration. BMJ. 2012; 345: e8343.

28. Kuriyama A, Honda M, Hayashino Y. Ramelteon for the treatment of insomnia in adults: a systematic review and meta-analysis. Sleep Med. 2014; 15: 385-92.

29. Kanji S, Mera A, Hutton B, et al. Pharmacological interventions to improve sleep in hospitalised adults: a systematic review. BMJ Open. 2016; 6: e012108.

30. Kishi T, Matsunaga S, Iwata N. Suvorexant for Primary Insomnia: A Systematic

For peer review only

For peer review only - http://bmjopen.bmj.com/site/about/guidelines.xhtml

Review and Meta-Analysis of Randomized Placebo-Controlled Trials. PLoS One. 2015; 10: e0136910.

31. Toi N, Inaba M, Kurajoh M, et al. Improvement of glycemic control by treatment for insomnia with suvorexant in type 2 diabetes mellitus. J Clin Transl Endocrinol. 2018; 15: 37-44.

32. Thomas AP, Hoang J, Vongbunyong K, et al. Administration of Melatonin and Metformin Prevents Deleterious Effects of Circadian Disruption and Obesity in Male Rats. Endocrinology. 2016; 157: 4720-4731.

33. Hung HC, Yang YC, Ou HY, et al. The association between self-reported sleep quality and overweight in a Chinese population. Obesity (Silver Spring). 2013; 21:486-92.

34. Younossi Z, Tacke F, Arrese M, et al. Global Perspectives on Nonalcoholic Fatty Liver Disease and Nonalcoholic Steatohepatitis Hepatology. 2019; 69:2672-2682.

35. Garfield V, Joshi R, Garcia-Hernandez J, et al. The relationship between sleep quality and all-cause, CVD and cancer mortality: the Southall and Brent REvisited study (SABRE). Sleep Med. 2019; 60: 230-5.

For peer review only

For peer review only - http://bmjopen.bmj.com/site/about/guidelines.xhtml

STROBE Statement—Checklist of items that should be included in reports of ***cohort studies***

	Item No	Recommendation	Page No
**Title and abstract**	1	(*a*) Indicate the study's design with a commonly used term in the title or the abstract	1
		(*b*) Provide in the abstract an informative and balanced summary of what was done and what was found	2
**Introduction**			
Background/rationale	2	Explain the scientific background and rationale for the investigation being reported	2-3
Objectives	3	State specific objectives, including any prespecified hypotheses	3
**Methods**			
Study design	4	Present key elements of study design early in the paper	5-7
Setting	5	Describe the setting, locations, and relevant dates, including periods of recruitment, exposure, follow-up, and data collection	5-7
Participants	6	(*a*) Give the eligibility criteria, and the sources and methods of selection of participants. Describe methods of follow-up	5
		(*b*) For matched studies, give matching criteria and number of exposed and unexposed	5
Variables	7	Clearly define all outcomes, exposures, predictors, potential confounders, and effect modifiers. Give diagnostic criteria, if applicable	5-7
Data sources/ measurement	8*	For each variable of interest, give sources of data and details of methods of assessment (measurement). Describe comparability of assessment methods if there is more than one group	5-7
Bias	9	Describe any efforts to address potential sources of bias	
Study size	10	Explain how the study size was arrived at	5
Quantitative variables	11	Explain how quantitative variables were handled in the analyses. If applicable, describe which groupings were chosen and why	
Statistical methods	12	(*a*) Describe all statistical methods, including those used to control for confounding	7
		(*b*) Describe any methods used to examine subgroups and interactions	7
		(*c*) Explain how missing data were addressed	
		(*d*) If applicable, explain how loss to follow-up was addressed	
		(*e*) Describe any sensitivity analyses	
**Results**			
Participants	13*	(a) Report numbers of individuals at each stage of study—eg numbers potentially eligible, examined for eligibility, confirmed eligible, included in the study, completing follow-up, and analysed	8
		(b) Give reasons for non-participation at each stage	
		(c) Consider use of a flow diagram	
Descriptive data	14*	(a) Give characteristics of study participants (eg demographic, clinical, social) and information on exposures and potential confounders	8-12
		(b) Indicate number of participants with missing data for each variable of interest	
		(c) Summarise follow-up time (eg, average and total amount)	
Outcome data	15*	Report numbers of outcome events or summary measures over time	8-12

For peer review only

Main results	16	(*a*) Give unadjusted estimates and, if applicable, confounder-adjusted estimates and their precision (eg, 95% confidence interval). Make clear which confounders were adjusted for and why they were included<br>(*b*) Report category boundaries when continuous variables were categorized<br>(*c*) If relevant, consider translating estimates of relative risk into absolute risk for a meaningful time period	8-12
Other analyses	17	Report other analyses done—eg analyses of subgroups and interactions, and sensitivity analyses	
**Discussion**			
Key results	18	Summarise key results with reference to study objectives	13
Limitations	19	Discuss limitations of the study, taking into account sources of potential bias or imprecision. Discuss both direction and magnitude of any potential bias	15
Interpretation	20	Give a cautious overall interpretation of results considering objectives, limitations, multiplicity of analyses, results from similar studies, and other relevant evidence	13-15
Generalisability	21	Discuss the generalisability (external validity) of the study results	
**Other information**			
Funding	22	Give the source of funding and the role of the funders for the present study and, if applicable, for the original study on which the present article is based	

*Give information separately for exposed and unexposed groups.

Note: An Explanation and Elaboration article discusses each checklist item and gives methodological background and published examples of transparent reporting. The STROBE checklist is best used in conjunction with this article (freely available on the Web sites of PLoS Medicine at http://www.plosmedicine.org/, Annals of Internal Medicine at http://www.annals.org/, and Epidemiology at http://www.epidem.com/). Information on the STROBE Initiative is available at http://www.strobe-statement.org.

For peer review only
